# Performance of Rigid and Soft Transfer Templates Using Viscous and Fluid Resin-Based Composites in the Attachment Bonding Process of Clear Aligners

**DOI:** 10.1155/2022/1637594

**Published:** 2022-02-12

**Authors:** Cristina Valeri, Angelo Aloisio, Stefano Mummolo, Vincenzo Quinzi

**Affiliations:** ^1^Department of Life, Health and Environmental Sciences, Postgraduate School of Orthodontics, Università degli Studi dell'Aquila, Via Piazzale Salvatore Tommasi 1, Abruzzo, L'Aquila 67100, Italy; ^2^Department of Civil, Environmental and Construction-Architecture Engineering, Università degli Studi dell'Aquila, Via Giovanni Gronchi n.18, L'Aquila 67100, Italy

## Abstract

**Objectives:**

The study aims at assessing the accuracy of the process of attachment bonding in aligner treatments. The analysis leads to the error estimation in the faithful reproduction of master model attachments using two types of transfer templates and two light-curing resin-based composites usually used in orthodontics.

**Methods:**

The authors have used two transfer templates made of two different materials. The first, named Leone-biocompatible thermoforming material hard/soft, has a lower Young's modulus and is labelled as soft, while the other, named Leone-biocompatible thermoforming material, is marked as rigid. The resin-based composites possess different mechanical and rheological properties. Specifically, Transbond™ XT Light Cure Paste Adhesive, 3M has a higher viscosity than the TetricEvoflow, Ivoclar Vivadent, a flowable nanohybrid composite. The authors attempt to estimate the performance ranking between the four possible couples obtained by combining the two light-curing resin-based composites and transfer templates. Each combination was repeated in six models and compared with twelve master models, resulting in 36 total samples. A 3-D laser scanner is used to generate a digital model of each model. The comparison between digital models is the base for a comparative assessment in terms of relative and absolute error. The relative error is estimated using scalar performance indicators ranging from 0 to 1, where 1 indicates the optimum matching. The absolute error estimated from the mean square error between the coordinates of each digital model yields the reproduction accuracy in micrometer. Furthermore, the authors attempted to assess the error distribution by evaluating the point-by-point difference between the digital models.

**Results:**

This analysis aims at localizing the sources of error in the considered models. The use of Transbond™ XT Light Cure Paste Adhesive, 3M with a rigid transfer template is always associated with significant accuracy and minor dispersion. However, in a few instances, using the soft template or the flowable resin-based composite can lead to bad performances. *Significance*. The data processing bestowed the following performance ranking from the first with lower reproduction error to the last characterized by the worst performance: (1) attachments bonding with rigid template and Transbond™ XT Light Cure Paste Adhesive, 3M, (2) attachments bonding with soft template and Transbond™ XT Light Cure Paste Adhesive, 3M, (3) attachments bonding with rigid template and TetricEvoflow, Ivoclar Vivadent, and (4) attachments bonding with soft template and TetricEvoflow, Ivoclar Vivadent.

## 1. Introduction

Clear aligners technique involves the sequential use of a transparent thermoplastic aligner, capable of guiding the teeth in the position previously planned on the virtual setup models, introducing small increments of 0.25–0.33 mm up to a maximum of 1 mm for each stage [1–5]. This technique is the common factor of an increasingly vast market offer that differs from thermoplastic materials' type, thicknesses, and stiffness [6–8]. This procedure can increase its effectiveness by using the CAD-CAM technology [2, 5, 7–18]. It can be particularly advantageous in patients with low levels of oral hygiene, periodontal disease, or a high predisposition to gingival recession. In all, those who cannot tolerate pain or have aesthetic needs [6, 9, 11, 19, 20]. Additionally, multidisciplinary pre-prosthetic or pre-surgical treatments can benefit from the use of aligners [19, 21]. Patient collaboration is an essential element to the success of the treatment [9, 19, 20, 22]. Clear aligners are not only appliances but also a technique. Therefore, knowledge of biomechanics is required to measure force-to-moment components that the aligner exerts on a single tooth [23]. There is a slight mismatch between tooth and aligner capable of generating a system of forces that starts the biological process of orthodontic movement [5, 10, 15, 16, 24]. The technique success depends on the contact areas between tooth and material, determined by tooth size and morphology [23], type and amount of movement desired, the internal surface of the aligner [24], the material properties (thickness, stiffness, and elastic modulus), and its fitting with the tooth [5–7, 10, 11, 13, 16]. In the scientific literature, there is a consensus on the dental movements that an aligner generates with predictability, like alignment and levelling of arches in mild and moderate crowding [6, 9, 15, 18, 25, 26], and endoinclination of teeth, especially of lower incisors [7, 9, 27].

Advances in materials, technology, careful planning of biomechanics, and using auxiliaries add more predictable movements to the technique [6, 9, 14, 20, 22, 24, 25, 28, 29]. A wide range of auxiliaries in the scientific literature increase the technique's effectiveness, but this article will focus only on the attachments. These differ in the following two categories: traditional and optimized. The traditional ones are rectangular or ellipsoidal and increase aligner retention. The optimized ones have various shapes with the aim of generating different orthodontic movements.

To the best of the authors' knowledge, the scientific literature lacks articles about the attachment bonding protocol, both in terms of the clinical steps to be carried out and the materials used. Similarly, it lacks scientific articles about transfer templates. The authors believe it is necessary to standardize the attachment bonding technique and improve the aligner biomechanics. The article's purpose is to evaluate the accuracy of the reproduction of attachments, comparing two transfer templates that differ in construction material and two resin-based composites that differ in viscosity.

The different mechanical properties of the transfer template and the rheological properties of the resin-based composites can lead to different accuracy in attachment bonding. Therefore, the authors compared the bonding performance by combining one resin-based composite with a specific transfer model. In detail, they used two resin-based composites, such as Tetric Evoflow (Ivoclar Vivadent) and Transbond™ XT Light Cure Paste Adhesive (3M), and two transfer templates that differ in construction material. One material has a higher Young's modulus than the other. Consequently, the former in the article will be labelled “rigid,” while the latter “soft.” The attachments that are bonding to the smooth tester models are compared to those present on the master model, resulting in 36 samples to be compared. The 3-D scan of each model allowed the direct comparison between the digital models generated by the scanner in terms of Cartesian coordinates of the acquired points.

The study is organized as follows: the second section presents the materials and methods. The third section compares the considered 36 samples obtained by using the two resin-based composites and the two typologies of transfer templates. The last section further discusses the results and the practical implications of this research.

## 2. Materials and Methods

**Figure 1:**
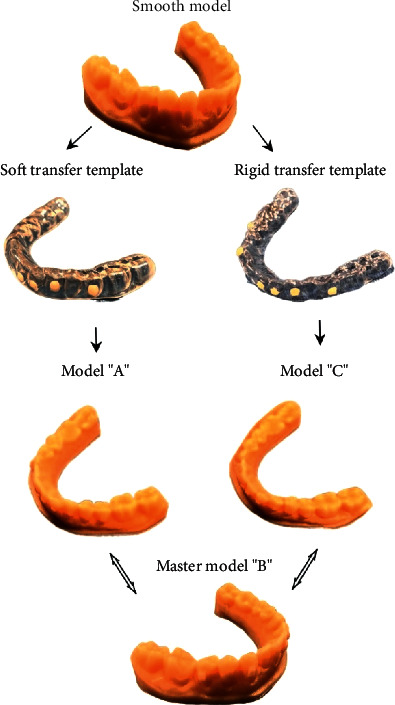
Illustration of the models to be compared.

This section describes the materials used and the methods followed in the current experimental investigation.  Figure 1 illustrates the comparison procedure. The authors compared a master model, labelled model B, with models A and C obtained by bonding the attachments using a soft and rigid transfer template, respectively. The attachments of models A and C are realized using two types of composite resins, whose commercial names are Transbond™ XT Light Cure Paste Adhesive, 3M and Tetric Evoflow, Ivoclar Vivadent.

Table 1 defines the 24 samples obtained by combining two transfer models and two resin-based composites. In total, the authors examined 36 models. Specifically, there are 12 master or B models, compared to 12 A models and 12 C models, detailed in Table 1, used for the attachment bonding. The following subsections detail the mechanical properties and intrinsic features of the A, B, and C models, the transfer templates, the attachment bonding process, and the model digitalization using a 3-D scanner.

### 2.1. Master Models and Tester Models

Twelve dental arches were chosen from the Leone Spa library. These had horizontal rectangular attachments, with a maximum horizontal extension of 4 mm, a maximum vertical extension of 2 mm, and a maximum height of 1 mm. Twelve master models, made of epoxy resin (E-Model Light), have been printed with a 3D-stereolithography printer (ULTRA, EnvisionTEC). In the same way, twenty-four tester models were produced. However, these are smooth models without attachments.

### 2.2. Transfer Template

Template A is made of a thermoplastic foil labelled “Leone-biocompatible thermoforming material hard/soft” of 2.0 mm thickness, 2200 MPa pre-forming elastic modulus in the rigid part, and 20 MPa in the soft one, thermoformed with a pressure forming device BioStar V, Scheu-Dental at 6 bar. After the thermoforming process, the template thickness is 1.5 ± 0.5 mm.

Template C is made of a thermoplastic foil labelled “Leone-biocompatible thermoforming material” of 0.5 mm thickness, 2200 MPa pre-forming elastic modulus, thermoformed with a pressure forming device Erkodent at 5 bar. After the thermoforming process, the template thickness is 0.15 ± 0.5 mm. Twenty-four templates were produced with material A and twenty-four with material B. One template is for one tester model to avoid introducing errors due to the material deformation after template removal following attachments bonding.

### 2.3. Attachment Bonding Process

A practitioner has bonded attachments without following the conventional bonding steps of resin-based composites. For this, the authors considered skipping the etching and adhesive bonding step because a resin model was used, not extracted teeth.

A practitioner has filled cells of the transfer templates with resin-based composite resin. Transbond™ XT Light Cure Paste Adhesive, 3M and Tetric Evoflow, Ivoclar Vivadent were chosen because they were usually used in orthodontic practice. They differ in density and compactness, allowing the authors to determine which guarantees a more faithful attachment transfer from the master to the tester models. The resin-based composite then was cured with the Valo Ortho curing lamp (wavelength 385–515 mn) following the manufacturer's instructions. This operation was repeated for 24 models following the same steps.

### 2.4. Models Scanning

The same practitioner has carefully scanned 36 models (24 smooth testers and 12 masters with attachments) using a CS 3600 intraoral scanner, Carestream Dental. The authors chose this scanner due to its trueness values [30–32].

The authors decided not to coat the models with powders or liquids because scanning-aid materials can be more effective on metallic surfaces than resin-like materials [33]. In addition, the manufacturer does not recommend it. Scanning was performed at room temperature of 23 ± 2°C following ISO 554 [34]. All models were scanned by a single operator, always in the same way.

### 2.5. Models Comparison

Accordingly, the comparison between models should be invariant to rigid rotations or translations, and it should focus on the sole informative portion of the model, excluding the upper part. Before processing the data, the authors selected the only informative part of the model corresponding to the teeth area. The authors compare the soft, the master, and the rigid templates in three steps. Initially, scalar numerical indicators invariant to rigid motions and sensitive to the sole deformation provide a synthetic assessment of the reproduction accuracy. In addition, these indicators support a ranking of performance between models. In a second step, the authors detail and quantify in micrometer the reproduction error of the master model by estimating the mean square error between the coordinates of the soft/rigid and master model. This analysis allows quantifying the error tolerance associated with each model and resin-based composite material. Ultimately, the authors attempt to localize the estimated discrepancies between models by representing a point-by-point difference between two models using a colour scale superposed to the B model 3-D representation. This study aims at localizing the sources of the detected differences and possibly understanding the physical reasons behind them. All numerical analysis and data processing have been carried out in Matlab.

#### 2.5.1. Synthetic Indicators

The similarity between models was estimated using two indicators for mutual validation, such as the rank correlation indicator (CI) [35] and the similarity index (SI) [36]. CI expresses the correlation between the point clouds of a reference model and those of the model to be validated. In particular, let **M**_*r*_ be the matrix that collects the coordinates of the points of the reference model and **M**_*v*_ the one that collects the coordinates of the points of the model to be validated. The correlation index has the following expression:

(1) $$\begin{array}{c} \text{CI}=\text{corr}\left(M_{r},M_{v}\right)=\frac{\text{vec }M_{r}\cdot \text{vec }M_{v}}{\left|\text{vec }M_{r}\right|\cdot \left|\text{vec }M_{v}\right|}, \end{array}$$

where (·) is the inner product, ‖ the vector norm, and vec the vectorialization operator. SI index derives from the field of image processing and quantifies the similarity between images. The mathematical formulation of the indicator is given in [36]. Both indicators range between 0 and 1, where 1 expresses the maximum correlation corresponding to identical models.

#### 2.5.2. Estimate of the Error Tolerance

The SI and CI are adimensional quantities and do not express the discrepancy between models in a specific unit of measure. The mean square error (MSE) in the *j* direction between the coordinates of the reference model (**M**_*r*,*j*_) and those of the model to be validated (**M**_*r*,*j*_^*∗*^), less than a rigid translation constant estimated from an ordinary least squares operator, can be written as follows:

(2) $$\begin{array}{c} {\text{MSE}}_{j}=E\left[{\left(M_{r,j}-M_{v,j}^{*}\right)}^{2}\right], \end{array}$$

where *E*(·) denotes the expected value operator.

#### 2.5.3. Localization of the Discrepancies between Models

The point-by-point distance between all coordinates of the master model and that to be validated can be written as follows:

(3) $$\begin{array}{c} d_{i}=\left|m_{i,r}-m_{i,v}^{*}\right|, \end{array}$$

where *d*_*i*_ is the Euclidean distance of a generic point *i* between the coordinates of the *i*-th point of the reference model (**m**_*i*,*r*_) and the coordinates of the *i*-th point of the model to be validated (**m**_*i*,*v*_^*∗*^), purified of the rigid body rotations and translations to the master model.

## 3. Results

This research aims to estimate the discrepancy between attachments present in the master model and those obtained using the rigid (Leone-biocompatible thermoforming material) and the soft template (Leone-biocompatible thermoforming material hard/soft) proposed to our attention by Leone SpA (Italy). For brevity, the authors will refer to the soft, the master, and the rigid templates as models A, B, and C, respectively. The superposition between the 3-D scan of the A-B and B-C models does not reveal a significant mutual discrepancy. Therefore, a quantitative estimate of the mutual differences is needed to accurately rank the A and C models' performance. Still, the two couples of models are not perfectly stackable. However, next to the possible shape modifications due to the A and C model deformations, a significant difference may depend on (i) rigid translations and rotations of the two compared models and (ii) the noninformative streaks on the upper part of the model.

Table 2 lists the values of the SI and CI for each of the twelve samples. The results are split into two sections, dedicated to the models realized using Transbond™ XT Light Cure Paste Adhesive, 3M and Tetric Evoflow, Ivoclar Vivadent composites. The table is divided into two sections in the vertical direction. The former refers to the comparison between the B and A model, labelled *S*_*A*,*B*_, while the second refers to the comparison between the B and C models, labelled *S*_*B*,*C*_. The chosen indicators exhibit consistent results. Higher SI always corresponds to higher CI and vice versa. Therefore, either the SI or the CI can be used for the performance ranking of the templates. Additionally, the variance of the indicators is minor in all cases. Consequently, the mean values of the indicators could be used for the model classification.

Table 2 lists the obtained correlation indicators. The direct inspection of Table 2 suggests the following ranking of performance. The first one is the most accurate, while the last one is the worst.

1. C-Transbond. The C model with attachments obtained using the rigid template and Transbond™ XT Light Cure Paste Adhesive, 3M composite exhibits the closest similarity with the master model. The SI and CI are approximately equal to 0.50 and 0.74, respectively.
2. A-Transbond. The A model with attachments obtained using the soft template and Transbond™ XT Light Cure Paste Adhesive, 3M composite exhibits close similarity with the master model. The SI and CI are approximately equal to 0.45 and 0.68, respectively.
3. C-Evoflow. The C model with attachments obtained using the rigid template and Tetric Evoflow, Ivoclar Vivadent composite is quite similar to the master model. The SI and CI are approximately equal to 0.41 and 0.65, respectively.
4. A-Evoflow. The A model with attachments obtained using the soft template and Tetric Evoflow, Ivoclar Vivadent composite exhibits the worst similarity to the master model. The SI and CI are approximately equal to 0.35 and 0.58, respectively.

The results in Table 2 do not provide the expected tolerance in micrometer associated with each sample, but they allow classifying the performances of the four considered models. The C-Transbond manifests the best similarity. The A-Transbond and C-Evoflow exhibit a good similarity with approximately equal performance indicators. The A-Evoflow sums two sources of error, the former derived from the soft template and the latter from a fluid composite (Tetric Evoflow, Ivoclar Vivadent). Therefore, A-Evoflow has the worst performance. The discussion of the consequences of this ranking within a decision-making process is discussed in the next section.

Table 3 lists the MSE in the three orthogonal directions (*x*, *y*, *z*) of each couple of models A-B and B-C in micrometer. The *x*, *y*, and *z* directions span the sample's width, depth, and height, as illustrated in all 3-D scan representations. The performance ranking of the MSE in Table 3 is in full accordance with that proposed in the previous subsection based on the SI and CI. The analysis of the results in Table 3 leads to the following considerations. The averaged discrepancy between models is always below 60 *μ*m in all samples and directions.

The MSE in the *x* and *y* direction is significantly higher than in the *z*-direction. Specifically, the MSE in the *z*-direction always ranges between 17 and 19 *μ*m with a minor dispersion. Conversely, the MSE in *x* and *y* directions span between 23 *μ*m in the C-Transbond model to 60 *μ*m in the A-Evoflow model. The performance ranking in terms of MSE matches with that based on synthetic indicators. The variance of the MSE in the *x*-direction is significantly higher than that in the *y*-direction, although the mean values are quite alike. The uncertainty in mirroring the B template in the *x*-direction is always higher than the *y*-direction, independently of the typology of composite or the rigidity of the transfer template. Still, using a more rigid template with a nonfluid composite, like Transbond™ XT Light Cure Paste Adhesive, 3M leads to a significant reduction of the MSE variance.

The results in the previous subsection revealed the estimated reproduction error in micrometer. However, the authors question whether the reproduction error is localized. The *d*_*i*_ value, defined in equation (3), is estimated in all model points in both the informative and noninformative portions. These values are represented as scatter plots in a colour map superposed to the B model. This representation allows detecting the sources of error based on the different colours of the plots.

**Figure 2:**
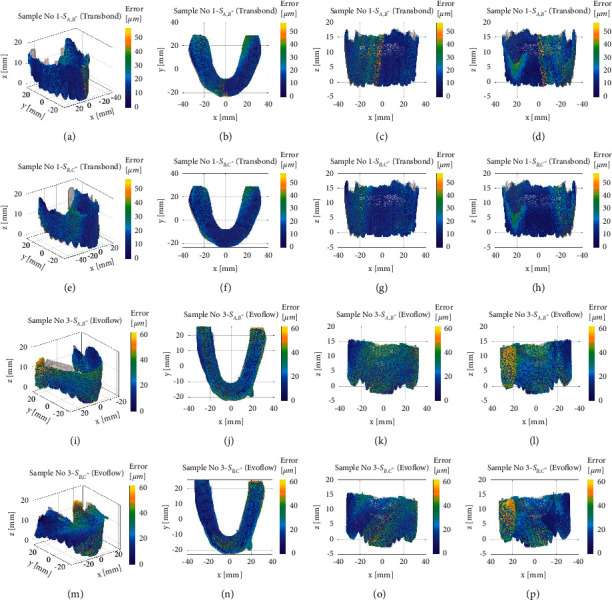
(a–d) Scatter plots of the relative distance between the A and B samples obtained with TetricEvoflow, Ivoclar Vivadent composite. The plot refers to sample no. 3. (e–h) Scatter plots of the relative distance between the B and C samples obtained with Transbond™ XT Light Cure Paste Adhesive, 3M composite. The plot refers to sample no. 3. (i–n) Scatter plot of the relative distance between the A and B samples obtained with TetricEvoflow, Ivoclar Vivadent composite. The plot refers to sample no. 3. (o–r) Scatter plot of the relative distance between the B and C samples obtained with TetricEvoflow, Ivoclar Vivadent composite. The plot refers to sample no. 3.

Figure 2 shows different views of the comparison between A-B Transbond, B-C Transbond, A-B Evoflow, and B-C Evoflow in selected cases. Specifically, Figure 2 shows the 3-D view, the overhead view, the front view, and the back view for each pair of specimens. The plotted Figures 2(a)–2(p) are selected as representatives of the four considered comparisons. In detail, Figures 2(a)–2(d) show the point-by-point distance between the B and C models realized with the Transbond™ XT Light Cure Paste Adhesive, 3M composite. As expected, the dominant colour of the map is blue, with a few regions of higher discrepancy in the lateral parts of the model. The variance is also minimal, with a few points with a relative distance higher than 40 *μ*m, prevalently in the noninformative part of the model.

Figures 2(e)–2(h) show the point-by-point distance between the A and B models in which only A model is realized with the Transbond™ XT Light Cure Paste Adhesive, 3M composite. The higher relative distance is mostly concentrated in the lateral part of the model, with higher dispersion than Figures 2(e)–2(h). The relative distance is higher in both the inner and outer part of the model, as evidenced by comparing subfigures (f) and (g).

Figures 2(i)–2(l) show the point-by-point distance between B and C models realized with the Tetric Evoflow, Ivoclar Vivadent composite. Figures 2(o)–2(p) show that the higher distance between models is not concentrated in the lateral part of the model, as observed in the models obtained with the Transbond™ XT Light Cure Paste Adhesive, 3M composite (Figures 2(e)–2(h)). The discrepancy is spread through the entire model. In the sample case shown in Figures 2(m)–2(p), the relative distance is higher in the frontal part, but other samples show diverse error localization. Therefore, the error in models realized with the fluid composite (like Tetric Evoflow, Ivoclar Vivadent) is more unpredictable.

Figures 2(i)–2(l) show the point-by-point distance between B and A models realized with Tetric Evoflow, Ivoclar Vivadent composite. Figures 2(i)–2(l) confirm the considerations referred to Figures 2(m)–2(p), although the relative distance is on average higher than any other sample. In conclusion, the analysis of the error distribution proves the following ones:

The use of a viscous resin-based composite, like Transbond™ XT Light Cure Paste Adhesive, 3M, leads to a higher error in the lateral parts of the model, while the front one is reproduced with great accuracy.

The use of a fluid resin-based composite, like Tetric Evoflow, Ivoclar Vivadent, is associated with a more uniform error distribution, scattered in all regions of the model.

Likely, the error distribution is independent of the typology of the model. The typology of the model (A or C) mainly affects the average value of this discrepancy, while the typology of resin-based composite influences the error distribution.

The specimens shown in Figure 2 are representative of sample cases. The authors wanted to witness the error distribution in all tested samples.

**Figure 3:**
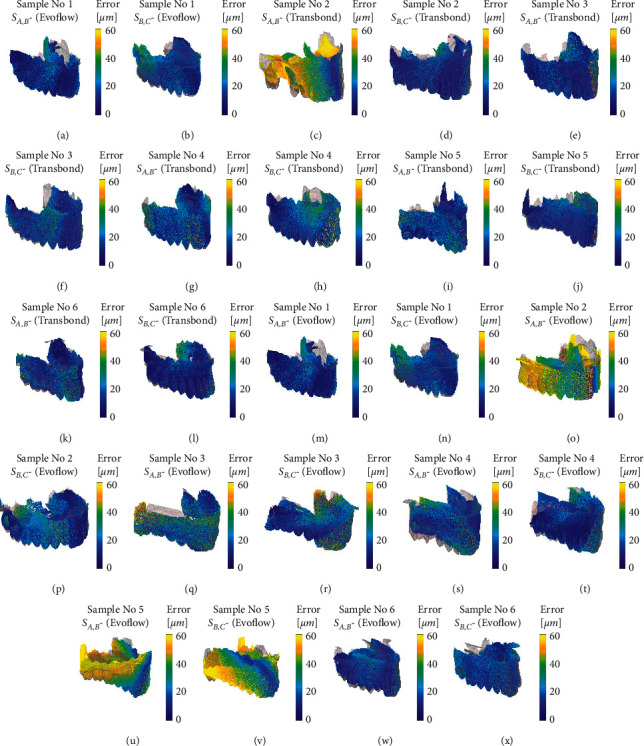
Scatter plot of the relative distance between the A-B and A-C samples in all considered cases.

Figure 3 collects the colour maps of all models, by limiting the representation to the sole 3-D view. Figure 3 confirms the above considerations and proves that the use of Transbond™ XT Light Cure Paste Adhesive, 3M with a rigid transfer template is always associated with significant accuracy and minor dispersion. However, in a few instances, the use of the soft template or the fluid resin-based composite can lead to bad performances, as shown in Figures 3(e), 3(o), 3(u), and 3(v). The authors only want to visually show with the scatter chart what was stated following the numerical analyses. They also decided to insert all the views of the model to give the reader a three-dimensional image. However, they are aware that the overhead view and the back view do not provide any clinical information.

## 4. Discussion and Results Interpretation

Attachments are essential auxiliaries in the aligner technique. They generate moment-to-force ratio components to guide orthodontic movements. These depend on material properties, amount of movement programmed in each aligner, tooth anatomy, and use of auxiliary accessories [5]. Attachments allow locating the orthodontic force acting on a particular tooth. It can therefore be the equivalent of the bracket in the fixed technique [24].

Conventional attachments have a rectangular or ellipsoidal shape and can increase the aligner's retention. However, Dasy et al. [37] show that ellipsoidal attachments cannot increase aligner's retention in all circumstances, like rectangular ones. They conclude the material of the aligner, rather than its thickness, changes the effect of the attachment.

Since the topic is not the objective of this article, the authors refer to the reading of the scientific paper by Daniele et al. [38] to allow the reader to learn more about the physicochemical and mechanical characteristics of the thermoplastic materials used in the production of orthodontic aligners. Optimized attachments have different shapes to increase the clinical effectiveness of the aligner in realizing more complex movements, such as rotation, intrusion, extrusion, or distalization of the upper molar [2, 15, 23, 24, 39–41]. Each attachment has an active surface oriented to provide the force in the desired direction [2] and create the appropriate moment-to-force ratio for each dental movement [4, 24].

The attachment design must minimize the contralateral components of the moment-to-force ratio, creating a counter-moment that moves the tooth in the opposite direction compared to the unwanted movement, such as intrusion in case of rotations and mesial tipping in distalization of the upper molars, and inclination in translations [23, 40–42].

The attachment position, rather than the shape, determines the effectiveness of the movement [3, 4]. The finite element analysis carried out by Barone et al. [5] demonstrates the effectiveness of the attachments and does not change substantially by varying their positioning on the crown of the tooth. At the same time, a slight variation occurs when changing their orientation.

Particular attention must be paid to the bonding process of optimized attachments, minimizing errors in the technique regarding the choice of the resin-based composite and the correct use of the transfer template from the model to the oral cavity. Incorrect positioning of the attachment results in wrong tooth movement and inadequate intensity of force [43]. There is also no consensus regarding the choice of the composite to be used to make the attachment. D'Antò et al. [44] compare the following three materials: flowable composite, conservative material, and orthodontic composite, coming to affirm that all three materials are suitable for producing attachments because they faithfully reproduce their shape, even if the orthodontic composite has higher values as overflow compared to the flowable one.

Barreda et al. [45] compare two bulk-fill resins (Filtek Z350 XT, 3M ESPE and Amelogen Plus TW, Ultradent Products Inc.) concluding that in the six months of treatment with aligners, there were alterations of the attachment surface while the shape was therefore not compromised. Thus, the clinical performance of both materials can be considered acceptable. Mantovani et al. [46] compare bulk-fill resin with flowable resin, stating that the former allows the aligner to fit better. Unfortunately, the attachment adhesion protocols are missing, so the choice is up to clinicians. Weckmann et al. [43] suggest the use of a flowable composite, preferably reinforced, and the two-step technique.

Our experimental investigation revealed the following aspects: the typology of resin-based composite causes more error than using a rigid or soft transfer template. Therefore, the practitioner should pay more attention to the composite selection rather than the rigidity of the transfer model. Accordingly, the mindful selection of a specific resin-based composite is more important than the transfer template material within a decision-making process.

The use of the Tetric Evoflow, Ivoclar Vivadent composite causes more error than the more viscous Transbond™ XT Light Cure Paste Adhesive, 3M. This phenomenon can depend on the bonding process and the effect of error propagation. The use of a resin-based flowable composite can be associated with incorporating air bubbles in the bonding process. In contrast, the use of a viscous resin cannot be associated with any material loss. Additionally, the effect of using a resin-based flowable composite on a soft, rigid template may determine the summation of the deformation bias caused by the use of two deformable materials.

The higher error associated with using a soft transfer template is related to the possible deformations induced during the bonding process from handling a deformable template.

It is challenging to predict the consequence of 60 rather than 20 *μ*m on the clear aligner performance. This investigation would require dedicated finite element investigations that are not the purpose of this research. Still, it must be remarked that the scientific literature has not proposed any quantitative correlation between the reproduction error in the clear aligners and its final performance.

Interestingly, the reproduction error is higher in the *x* and *y* directions, compared to the *z*-direction. This fact depends on the model geometry, characterized by a limited vertical development in the vertical direction, lower than 20 cm, compared to the in-plane dimension. The consequences of the vertical deformation are therefore more limited in the vertical direction. Between the *x* and *y* directions, the *x* one exhibited the highest error. This fact also depends on the model geometry. The largest dimension is along the *x*-direction (≈80 mm) since the *y*-direction is approximately 40 mm. Therefore, given the same material deformation, the consequent displacement is higher along the higher dimension.

The use of the Transbond™ XT Light Cure Paste Adhesive, 3M causes a higher error on the model sides, while that on the front is minor. Conversely, the use of the Tetric Evoflow, Ivoclar Vivadent is associated with a uniformly distributed error. This fact is challenging to understand, and the authors did not find a convincing physical reason behind it.

A possible cause could lie in the scanning accuracy of the intraoral scanner. The less accurate reproduction of the attachments may be present in models with asymmetries between the right and left sides of the arch. This inaccuracy could be because the intraoral scanner constructs the image using mirror reproduction [33].

From the data of this study, the soft template could be used as the rigid one due to the minimal error introduced in attachments reproduction. In addition, the softness of the material allows the clinician a more accessible and more immediate removal. Conversely, removing the rigid template, not allowing large deformations, increases the risk of removing the bonded attachments and lengthens the working time.

## 5. Conclusions

The data processing bestowed the following performance ranking from the first with lower reproduction error to the last characterized by the worst performance: (1) C-Transbond, (2) A- Transbond, (3) C-Evoflow, and (4) A-Evoflow.

Interestingly, the use of resin-based composites with different rheology has more consequences than using a rigid or soft transfer template. Therefore, within a decision-making process, the practitioner must pay more attention to the composite selection rather than the typology of the transfer model. The reproduction error approximately spans between 20 *μ*m and 60 *μ*m. However, the authors cannot affirm whether errors in these ranges can have significant consequences on the performance of a clear aligner.

Two sources of uncertainties mainly limit the validity of the research outcomes. The first is considering a limited number of samples. The second source of uncertainty is not including the reproducibility of the adhesion process, possibly related to the operator-induced error. Therefore, future research efforts will aim at extending the sample data and including additional operators within the process. Furthermore, the authors will aim at assessing the correlation between the reproduction error due to the use of a particular transfer template or resin-based composite and the clinical performance of clear aligners.

## Figures and Tables

**Table 1:** Combination grid of the samples obtained by using the two resin-based composites and the two transfer templates. The table indicates the number of samples associated with each combination.

Number of samples		
Typology	Transbond	Evoflow
Model A	6	6
Model C	6	6

**Table 2:** Estimate of the similarity and correlation indicators between the samples A-B and B-C in the case of two composite materials, labelled transbond and evoflow, respectively.

Composite typology	Sample no.	Sample A-B		Sample B-C	
		SI	CI	SI	CI
Transbond	1	0.434	0.673	0.451	0.668
Transbond	2	0.211	0.368	0.532	0.801
Transbond	3	0.485	0.736	0.569	0.815
Transbond	4	0.488	0.757	0.390	0.621
Transbond	5	0.554	0.783	0.509	0.752
Transbond	6	0.521	0.782	0.574	0.820

Mean		0.449	0.683	0.504	0.746
Variance		0.015	0.025	0.005	0.007

Evoflow	1	0.462	0.805	0.530	0.790
Evoflow	2	0.241	0.480	0.496	0.772
Evoflow	3	0.254	0.428	0.412	0.608
Evoflow	4	0.467	0.715	0.467	0.721
Evoflow	5	0.203	0.303	0.187	0.296
Evoflow	6	0.493	0.768	0.374	0.714

Mean		0.353	0.583	0.411	0.650
Variance		0.018	0.043	0.015	0.034

**Table 3:** Mean square error between the A-B and A-C models realized using the two composite materials, Transbond and Evoflow, respectively.

Composite typology	No.	Mean square error A-B (*μ*m)			Mean square error B-C (*μ*m)		
		*x*	*y*	*z*	*x*	*y*	*z*
Transbond	1	30.322	53.638	19.586	31.502	54.391	19.408
Transbond	2	125.508	46.390	17.795	14.687	45.568	17.435
Transbond	3	17.016	55.458	19.093	10.897	46.486	19.416
Transbond	4	20.289	50.228	20.065	37.971	59.983	19.335
Transbond	5	28.486	45.798	20.942	27.498	48.697	21.519
Transbond	6	21.054	45.594	19.317	15.342	41.808	19.315
Mean		40.446	49.518	19.466	22.983	49.489	19.405
Variance		1762.482	18.357	1.101	118.176	43.635	1.673
Evoflow	1	13.755	44.713	18.213	14.556	46.882	19.889
Evoflow	2	121.817	66.324	17.207	11.414	50.994	16.524
Evoflow	3	63.039	65.588	13.612	42.326	60.933	16.641
Evoflow	4	30.653	52.916	18.412	29.332	52.308	18.414
Evoflow	5	118.264	61.706	17.811	115.805	61.378	18.456
Evoflow	6	10.221	44.417	19.667	15.017	47.473	20.542

Mean		59.625	55.944	17.487	38.075	53.328	18.411
Variance		2540.468	100.426	4.266	1586.428	40.990	2.685

## Data Availability

Data are available from the corresponding author upon reasonable request.

## Additional Points

(i) Accuracy evaluation of attachment bonding process in aligner treatments using different materials for transfer templates and composite materials. (ii) Rigid templates with more viscous composites yield lower reproduction error than a soft template with flowable composites. (iii) The averaged discrepancy between models is consistently below 60 *μ*m in all samples and directions. (iv) Using a viscous resin-based composite leads to a higher error in the lateral parts of the model compared to the front side. (v) Using a fluid resin-based composite is associated with a more uniform error distribution.
